# Comparison between Mechanical Properties and Joint Performance of AA 2024-T351 Aluminum Alloy Welded by Friction Stir Welding, Metal Inert Gas and Tungsten Inert Gas Processes

**DOI:** 10.3390/ma17133336

**Published:** 2024-07-05

**Authors:** Miodrag Milčić, Damjan Klobčar, Dragan Milčić, Nataša Zdravković, Aleksija Đurić, Tomaž Vuherer

**Affiliations:** 1 Faculty of Mechanical Engineering, University of Nis, Aleksandra Medvedeva 14, 18000 Niš, Serbia; miodrag.milcic@masfak.ni.ac.rs (M.M.); dragan.milcic@masfak.ni.ac.rs (D.M.); natasa.zdravkovic@masfak.ni.ac.rs (N.Z.); 2 Faculty of Mechanical Engineering, University of Ljubljana, Aškerčeva 6, 1000 Ljubljana, Slovenia; damjan.klobcar@fs.uni-lj.si; 3 Faculty of Mechanical Engineering, University of East Sarajevo, Vuka Karadžića 30, 71123 Sarajevo, Bosnia and Herzegovina; aleksija.djuric@ues.rs.ba; 4 Faculty of Mechanical Engineering, University of Maribor, Smetanova 17, 2000 Maribor, Slovenia

**Keywords:** AA2024-T351, friction stir welding (FSW), tungsten inert gas (TIG), metal inert gas (MIG), mechanical properties

## Abstract

The aim of this work is to study joining Al 2024-T3 alloy plates with different welding procedures. Aluminum alloy AA 2024-T351 is especially used in the aerospace industry. Aluminum plates are welded by the TIG and MIG fusion welding process, as well as by the solid-state welding process, friction stir welding (FSW), which has recently become very important in aluminum and alloy welding. For welding AA2024-T35 with MIG and TIG fusion processes, the filler material ER 4043—AlSi_5_ was chosen because of reduced cracking. Different methods were used to evaluate the quality of the produced joints, including macro- and microstructure evaluation, in addition to hardness and tensile tests. The ultimate tensile strength (UTS) of the FSW sample was found to be 80% higher than that of MIG and TIG samples. The average hardness value of the weld zone of metal for the MIG- and TIG-produced AA2024-T3511 butt joints showed a significant decrease compared to the hardness of the base metal AA2024-T351 by 50%, while for FSW joints, in the nugget zone, the hardness is about 10% lower relative to the base metal AA2024-T3511.

## 1. Introduction

Aluminum constructions find frequent applications in various transportation sectors, notably in automotive engineering, railway vehicle manufacturing [1], ship construction, aviation [2], and space technology, thanks to the favorable mechanical properties and lightweight nature of aluminum alloys. The welded assemblies of automobiles, locomotives, marine vessels, aircraft, and spacecraft, comprised of diverse aluminum alloys, are primarily interconnected using fusion welding methods such as metal inert gas (MIG) [3,4] and tungsten inert gas (TIG) welding [5].

The fusion welding techniques adeptly bond materials with favorable weldability characteristics, defined by the ability to create defect-free welded joints. The weldability of aluminum alloys is influenced by various factors including their affinity to oxygen, high thermal expansion and conductivity, significant shrinkage upon solidification, and notable hydrogen solubility in the liquid phase, a trait that diminishes considerably during the solidification process. By welding aluminum alloys, the mechanical properties and resistance to corrosion in the HAZ decrease; porosity, hardening and solidification cracks appear. Aluminum alloys are welded with additional material with increased Si or Mg content. The appearance of defects in the area of welded metal is also affected by poorly prescribed technology of the welding procedure, which reduces the reliability of the welded structure. Remarkably, the typical imperfections associated with traditional welding methods, including solidification cracking, oxidation, distortion, and porosity, are absent in friction stir welding (FSW) applications [6,7,8]. 

FSW is a solid-state welding process that uses the heat generated between the tool and base metals. FSW shows various advantages over the fusion welding process [9,10], such as reducing manufacturing time and minimal deformation and distortion of the joints [11]. In the last 20 years, friction stir welding has been increasingly applied to weld various aluminum alloys [12,13,14,15].

Laser and electron beam welding are used to weld parts of light structures made of aluminum alloys [16]. 

Aluminum alloy 2024, which is known for age-hardening, is part of the 2XXX series of alloys where copper is the primary alloying element. These alloys achieve mechanical properties that can be comparable to carbon steels due to the formation of CuAl_2_ particles during natural or artificial aging processes. Despite their excellent strength, these alloys exhibit poor corrosion resistance, which is why they are often coated with pure aluminum to enhance their corrosion protection. They are predominantly employed in the aviation industry owing to their high strength and excellent fatigue characteristics. Additionally, the incorporation of elements like magnesium and lithium reduces the specific density and enhances the performance of aluminum alloys for aerospace applications [17].

Typically, 2XXX series alloys exhibit poor weldability when using fusion welding techniques such as MIG or TIG due to their high susceptibility to cracking. Consequently, friction stir welding (FSW) is predominantly utilized for joining these alloys [18,19]. This study presents a comparative analysis of the structural and mechanical properties of butt-welded joints produced by MIG, TIG, and FSW methods. The mechanical properties of MIG and TIG joints were compared with those of FSW joints fabricated under optimal parameters [18,19]. The evaluation of the quality of the welded joints was conducted through visual inspection, macro- and microstructural analysis, hardness measurements of the welded joint, and tensile property testing.

## 2. Materials and Methods

### 2.1. Material

AA2024-T351 aluminum alloy rolled sheets were welded in a butt-joint configuration using TIG, MIG, and FSW techniques. The 8 mm thick AA2024-T351 sheets were cut into plates measuring 300 mm in length and 125 mm in width for welding with the aforementioned techniques. The chemical and mechanical properties of the AA2024-T351 alloy, based on standard specifications, are provided in Table 1 and Table 2. 

### 2.2. Fusion Welding of AA2024-T351

The AA2024-T351 plates were fusion-welded using two different techniques: TIG and MIG welding processes. The TIG arc was shielded with argon gas (ISO 14175-I1-Ar 5.0), while the MIG arc was shielded with a gas mixture of Argon + He (ISO 14175-I3-ArHe-30). In this experiment, a Fronius Transpuls Synergic 4000 direct-current electrode-positive (DCEP) MIG welding machine and a Fronius Magic Wave 4000 Job G/F direct-current electrode-negative (DCEN) TIG welding machine (Fronius, Wels, Austria) were used in producing the welds. The chemical properties of the filler material used during welding are given in Table 3.

The welding parameters for MIG and TIG butt welding of aluminum alloy 2024-T351 are provided in Table 4, where the parameter η is thermal efficiency.

### 2.3. FSW of AA2024-T351

**Figure 1:**
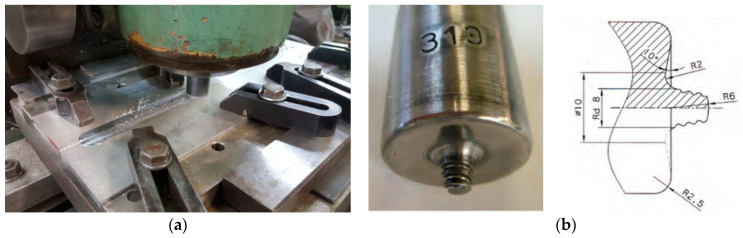
(**a**) Conventional milling machine for FSW and (**b**) geometry of FSW tool.

FSW of AA2024-T351 was carried out using a conventional vertical milling machine (Figure 1a). The welding tool had a tapered threaded cylindrical shape, with its dimensions and geometry displayed in Figure 1b. The FSW tool was made from 55CrMo_8_ tool steel and was heat-treated to achieve a hardness of 50 HRC.

The dimensions of the welding samples were 500 mm × 65 mm × 6 mm. Before welding, an austenitic plate was utilized as the base plate under the welding plate. The length of the weld was approximately 400 mm.

The most critical parameters in FSW are the welding speed and the tool’s rotational speed. Experimental research was conducted with a constant tool rotational speed, while varying the welding speed (Table 5).

By investigating the mechanical properties of joints welded through FSW for the welding parameter of the number of revolutions of the tool/welding speed (n/v), A-I (750/73), B-II (750/116), and C-III (750/150), the optimal parameters were found: tool speed n = 750 rpm and welding speed v = 116 mm/min [18,19]. In the following, a comparison of the mechanical and structural properties of butt welds made by MIG and TIG welding will be compared with FSW welds made with welding parameters n = 750 rpm and v = 116 mm/min.

### 2.4. Characterization of AA2024-T351 Welds

Test specimens for macro- and microstructural analysis, including tensile testing and hardness testing, were prepared from the welded samples by water jet cutting. The microstructure was investigated on the cross-section of the samples following standard metallographic preparation and etching with Keller’s reagent. A Leica Q500MC optical microscope (LM) (Leica, Wetzlar, Germany) was utilized to analyze the microstructure of the welded joint. Additionally, tests were conducted using a JOEL JSM-6610LV scanning electron microscope (SEM) (JOEL, Tokyo, Japan). 

**Figure 2:**
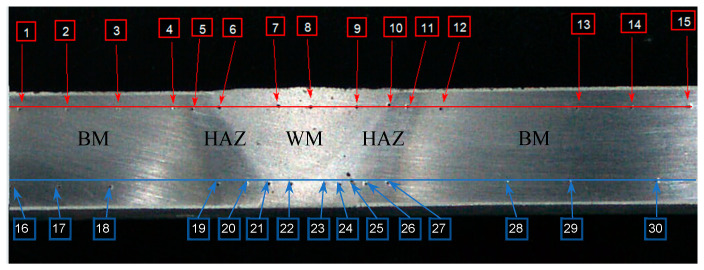
Hardness measurement points of the joint.

The Vickers hardness measurement of MIG and TIG welded joints was performed on a Willson VH1150 hardness measuring device (Buehler, Lake Bluff, IL, USA). According to the standard procedure, hardness measurements were made along two horizontal directions near the butt and near the root of the weld with three hardness measurements each in the base metal (BM), three in the heat-affected zone (HAZ), and three in the weld metal (WM). Figure 2 show the arrangement of hardness measurement points in tested welded joints. Measurement points are marked with numbers 1–30 and apply to MIG and TIG welded joints.

**Figure 3:**
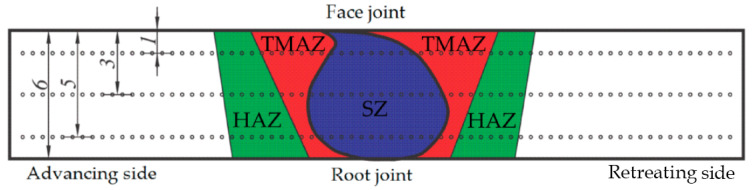
Microhardness measurement scheme with characteristic zones of welded joint (SZ—stir zone, TMAZ—thermomechanically affected zone, HAZ—heat-affected zone).

Vickers hardness measurement of joints welded through FSW was conducted using the HVS-1000 micro Vickers hardness tester (TIME, Beijing, China). The hardness profile was analyzed along three horizontal directions: the weld face (1 mm from the face of the welded joint), the weld center (3 mm from the face of the welded joint), and the weld root (5 mm from the face of the welded joint) (Figure 3). The distance between the indentations was 0.5 mm.

Tensile properties were assessed at room temperature using a Shimadzu AG-X 300 kN tensile tester (Shimadzu, Kyoto, Japan). Test specimens, defined by the ASTM E8M standard [21] and obtained from welded samples perpendicular to the weld joint, were employed. Specimens were cut from the welded samples using the water jet cutting process.

## 3. Results and Discussion

### 3.1. Visual Inspections

Following the welding processes, the welded samples were evaluated through visual inspection, metallographic tests, Vickers hardness tests, and tensile tests to compare the effects of the welding processes on the quality of joining 8 mm AA2024-T351 in similar butt welds.

**Figure 4:**
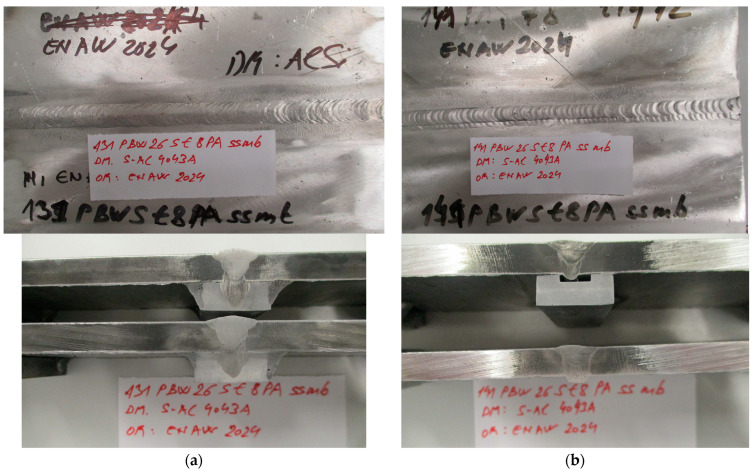

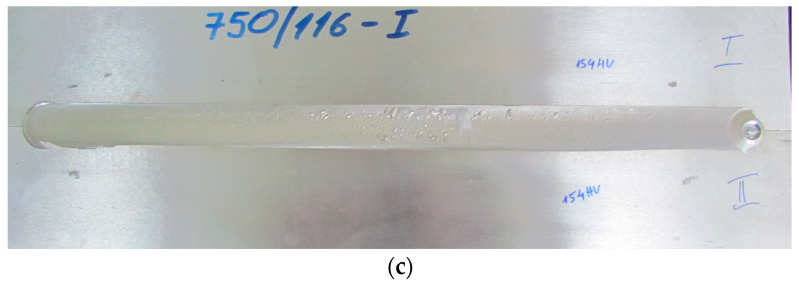
Face side of the welded joint with (**a**) MIG; (**b**) TIG; (**c**) FSW process.

Figure 4 illustrates the face side of a welded joint. The welded joint was made with a backing material (Figure 4a,b).

### 3.2. Macro- and Microscopic Examinations

**Figure 5:**
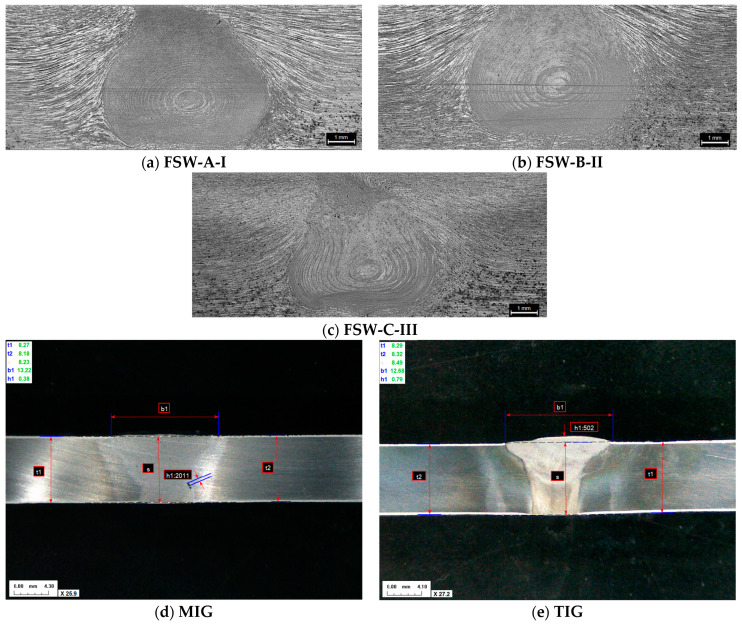
Macrostructure of a welded joint.

Figure 5 presents the macrostructure of the AA 2024-T351 butt joints, welded using FSW (Figure 5a–c), MIG (Figure 5d), and TIG (Figure 5e) techniques, observed on the cross-section of the weld axis. In the FSW joins, there are no defects such as tunnels or cracks. The MIG and TIG joint has a regular symmetrical shape without apparent defects such as cracking, undercutting, and porosity.

The macrostructure of FSW welds clearly indicates three characteristic zones: the stirred zone (SZ), the thermomechanically affected zone (TMAZ), and the heat-affected zone (HAZ). Also, in MIG and TIG welded joints, characteristic zones are observed: the weld metal zone (WM) and the heat-affected zone (HAZ). The heat-affected zone (HAZ) of the TIG welded joint is wider than the heat-affected zone of the MIG welded joint, due to the greater amount of heat input.

**Figure 6:**
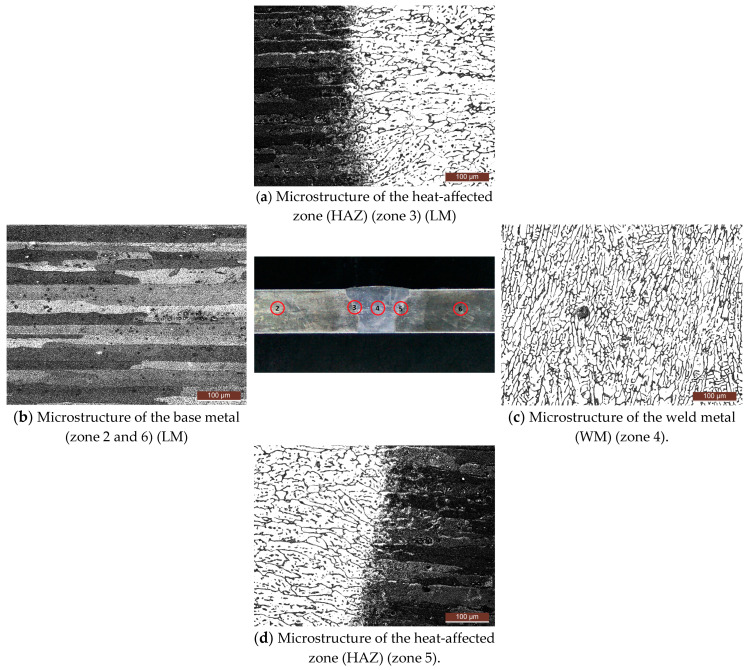
Appearance of cross-section of the specimen with view of microscopic examination zones—MIG [22].

The microstructure of the MIG welded joint is given in Figure 6.

For the microstructure of the zone between the HAZ and the weld metal (WM) (zone 3–Figure 6a), precipitate particles are observed in the weld metal up to the HAZ, separated by the grain boundaries and in the grain to a certain extent with a narrow columnar orientation. In HAZ, particles of intermetallic phases (IMPs) are separated by grain boundaries, and larger IMP particles are also present. In the microstructure of the weld metal (zone 4—Figure 6c), precipitate particles separated by the grain boundaries, and, in places, also in the grain, can be observed. The grains are of different sizes and have a dendritic orientation.

**Figure 7:**
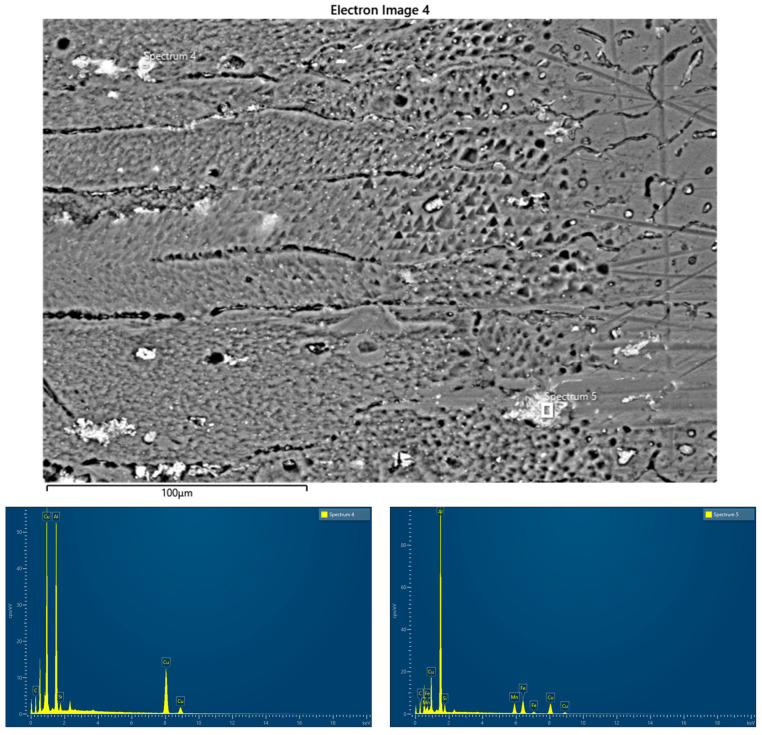
EDS spot analysis of MIG joint sample (SEM).

Figure 7 shows the EDS analysis of zones 3 and 4 (Figure 6a). Table 6 shows the results of the element concentration percentages.

Single precipitates were analyzed with EDS to determine their chemical composition. EDS analysis was performed on two different points of the weld metal zone (WM) and heat-affected zone (HAZ). In point 4 (the silverish spot), the atomic percentage of the element Cu is 55% (highest), the atomic percentage of the element Al is 43% (second highest), and that of Si is 2.15%. Therefore, point 4 has the element Cu as the majority element for the silvery spot, possibly from the AA2024 parent metal. In point 5, the atomic percentage of the element Al is 55% (highest), the atomic percentage of the element Cu is 20.2% (second highest), that of Fe id 13% (third highest), that of Mn is 8.6% (fourth highest), and that of Si is 3.23%. It was found that point 4 consists of the Fe_m_Al_n_ compound, and the Cu element was normally found as the alloying element for AA2024.

**Figure 8:**
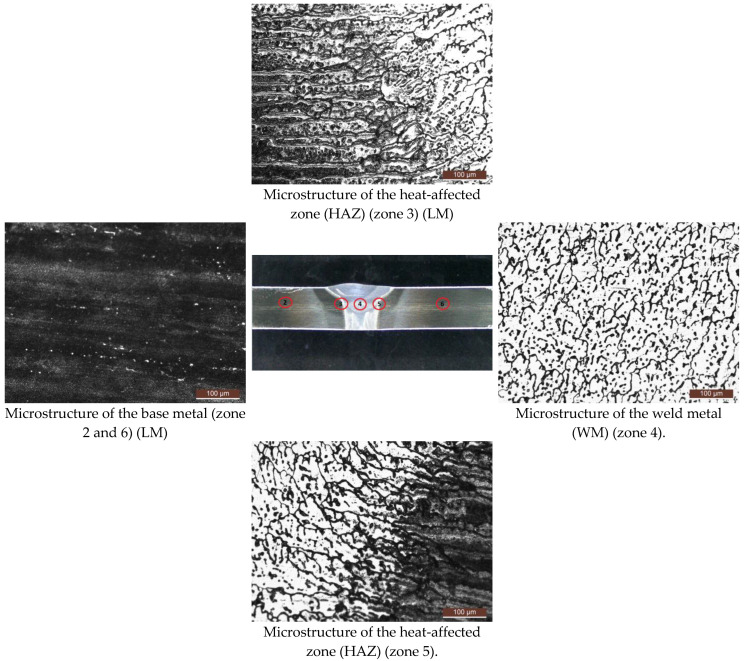
Appearance of cross-section of the specimen with view of microscopic examination zones—TIG.

The microstructure of the TIG welded joint is given in Figure 8.

**Figure 9:**
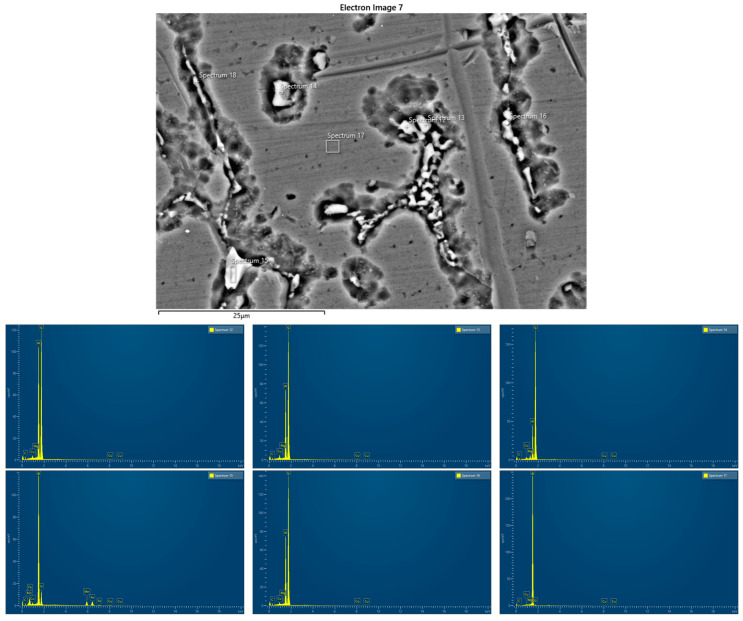
EDS spot analysis of TIG joint sample (SEM).

The microstructure of the base material AA2024-T351 (zones 2 and 6) has an elongated grain in the rolling direction with separated large particles of IMP precipitate. In the microstructure of the zone between the weld metal and HAZ (zone 3 and 5), sediment particles can be observed at the boundaries and inside the grain, and the grain has an orientation in the direction of rolling. In the microstructure of the weld metal zone, sediment particles of different sizes are observed at the boundaries and in the grain itself. The grain has a dendritic orientation. Figure 9 shows different points of the TIG welded sample and Table 7 shows the results of the element concentration percentages.

EDS analysis was performed on five different points of the weld metal zone. Point 17 is the main matrix, and the amount of Al measured is high. In points 12, 13, 14, and 16 (the silverish spot), the atomic percentage of the element Si is between 62% and 81% (highest), the atomic percentage of the element Al is between 16 and 35% (second highest), that of Cu is between 0.74% and 1.27%, and that of Mg is between 0.21% and 1.72%. It was shown that in points 12, 13, 14, and 16, Al-Si is an alloy from ER4043, which is from the Al-Si alloy group. The atomic percentages of Cu and Mg elements in WM were relatively high due to the IMC (intermetallic compound) interaction that occurred during the solidifying process, and this produced Al_m_Cu_n_ and Al_m_Mg_n_ compounds. It was found that in this region, the elements from a parent metal such as Cu and Mg were solidified together with the elements presented in the weld metal, producing IMCs. In point 15, the content of Al is 59.4% (highest), that of Fe is 15% (second highest), Mn is 13% (third highest), Si is 10% (fourth highest), and Cu is 2.6%. The atomic percentages of Fe and Mn elements in WM were relatively high due to the IMC interaction that occurred during the solidifying process, and this produced Al_m_Fe_n_ and Al_m_Mn_n_ compounds.

**Figure 10:**
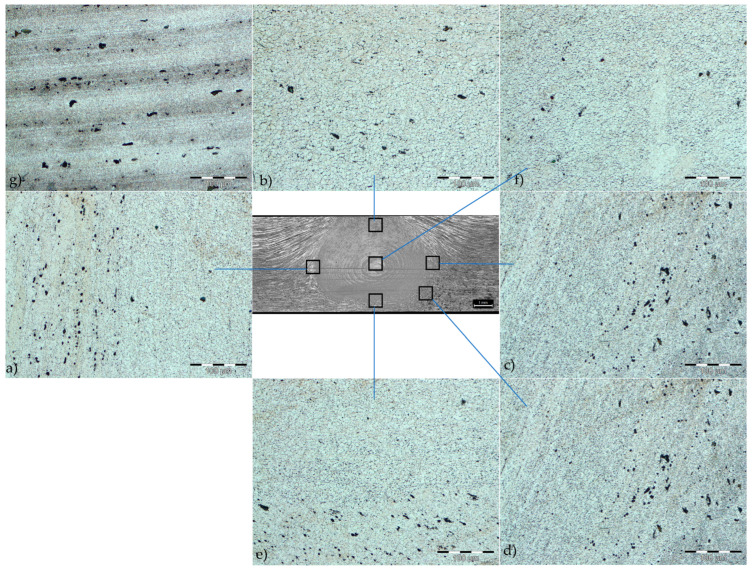
The microstructure of FSW joint: (**a**–**e**) interface between the SZ and the TMAZ; (**f**) SZ; (**g**) BM.

The microstructure of the FSW welded joint is given in Figure 10.

The HAZ is poorly defined. In different areas of the joint (Figure 10a–e) of the thermomechanically affected zone (TMAZ), grains of different sizes and orientations were obtained. The grain size in the TMAZ decreases towards the nugget region. The structure in the nugget (stir zone) is very fine-grained. A significant number of dark spots, which are sediment particles, can be observed in the TMAZ. The microstructure of the nugget is characterized by very small, equiaxed grains (Figure 10f), which is attributed to dynamic recrystallization.

### 3.3. Tensile Testing

Tensile tests were conducted on specimens cut from AA2024-T351 welds created using TIG, MIG, and FSW techniques. These tests aimed to determine tensile properties such as ultimate tensile strength (UTS), yield strength (YS), and elongation (E%). The tensile test results are given in Table 8. The place of fracture of the tested specimens obtained by all welding procedures are in the weld metal for TIG and MIG welded specimens, i.e., in the stir zone for the FSW specimen.

**Figure 11:**
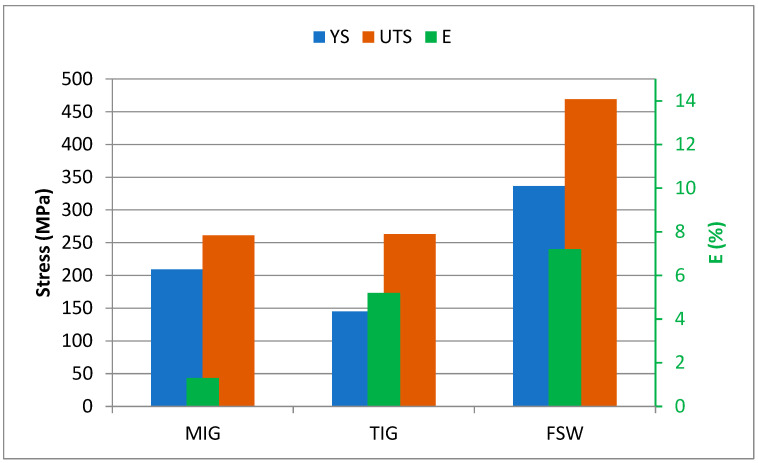
Ultimate tensile strength, yield strength, and elongation percentage of the similar AA2024-T351 butt joints welded using different techniques.

The comparative results of the tension test are shown in Figure 11.

It was found that the tensile properties (UTS and YS) and percentage elongation (E) of the welded tensile specimens by friction stir welding are better than the properties of the welded tensile specimens obtained by conventional welding methods, i.e., TIG and MIG welding [23,24,25]. The joint efficiency, which is the ratio of the tensile strength of the welded joint to the tensile strength of the base metal, is 97% for friction stir welding compared to 54% and 55%, respectively, for the MIG and TIG welding processes.

### 3.4. Hardness Distribution

**Figure 12:**
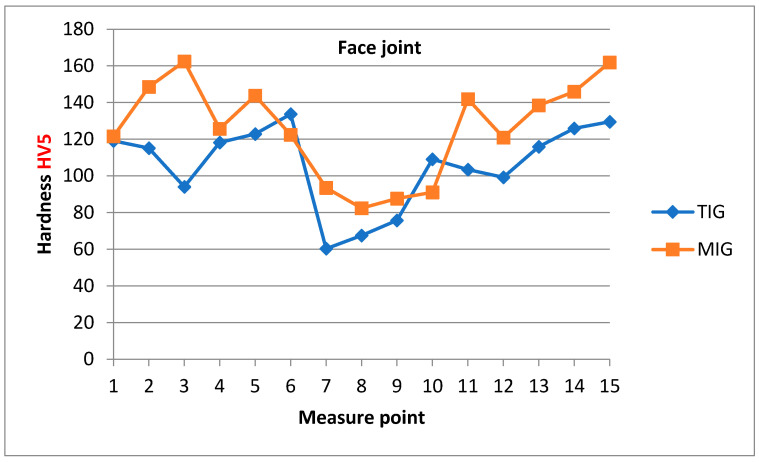
Hardness distribution of MIG and TIG butt joints obtained by measuring near the weld face.

**Figure 13:**
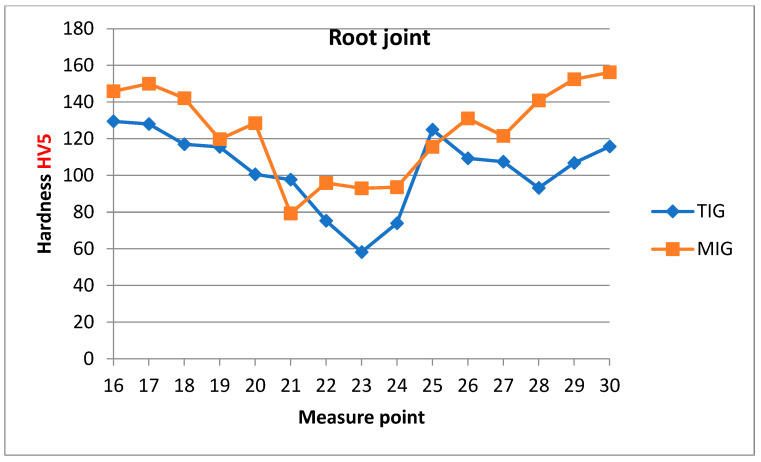
Hardness distribution of MIG and TIG butt joints obtained by measuring near the root of the weld.

Figure 12 shows the Vickers hardness profile of the cross-section of MIG and TIG welded joints measured near the weld face, while Figure 13 shows the hardness profile of MIG and TIG welded joints measured near the root of the weld. For an MIG welded joint, the average hardness in the weld metal zone near the weld face is 88 ± 5.5 HV, and for a TIG welded joint, the average hardness in the weld metal zone near the weld face is 68 ± 7.7 HV. For the MIG welded joint, the average hardness in the weld metal zone near the weld root is 94 ± 1.5 HV, and for the TIG welded joint, the average hardness in the weld metal zone near the weld root is 69 ± 9.5 HV. The hardness of TIG welds in the weld metal zone is lower than that of MIG welds due to the greater amount of heat introduced during the welding process. The hardness of both MIG and TIG welded joints in the HAZ is higher than in the weld metal, and lower in relation to the hardness of the base metal [16,17].

**Figure 14:**
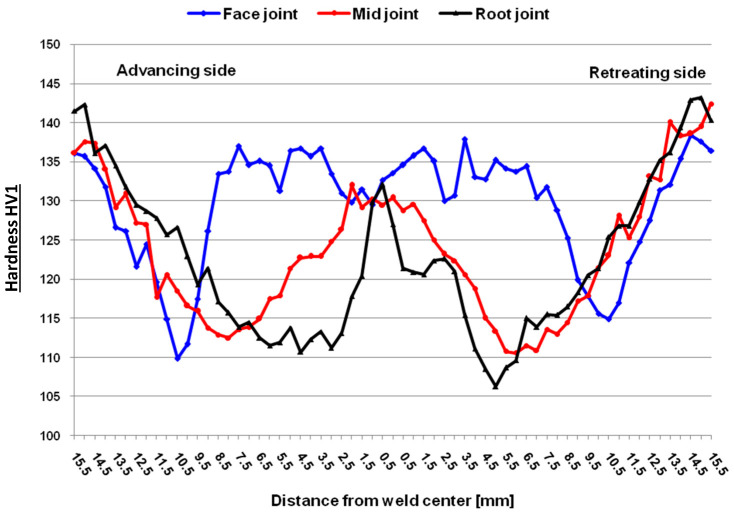
Hardness distribution of FSW butt joint obtained by measuring in the middle, near the face and root of the seam.

In the FSW welded joint (Figure 14), the highest hardness due to recrystallization is in the stir zone (SZ). The lowest hardness is observed in the HAZ (about 110 HV), and then the hardness recovers in this region and increases to the level of the base metal hardness.

**Figure 15:**
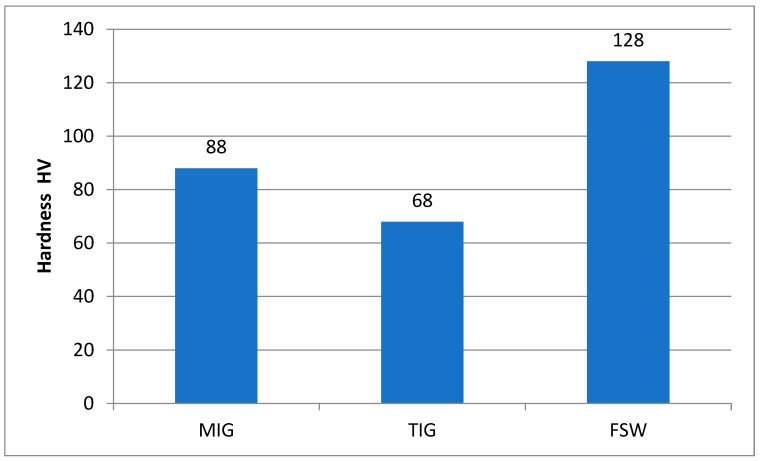
A comparative assessment among hardness levels at the WZ.

For a better understanding, a comparative graph of the hardness in the weld metal zone of MIG and TIG and in the stir zone of the FSW welding process is presented in Figure 15. The highest hardness is in the mixing zone of the FSW sample, followed by the weld metal zone of the MIG and TIG samples, respectively.

## 4. Conclusions

The influence of three joining techniques, TIG and MIG as fusion welding processes and FSW as a solid-state welding process, on the quality and properties of butt welds of aluminum alloy AA2024-T351 was investigated. Based on the test results, the following conclusions can be drawn:

1. The average hardness value for FSW joints in the stir zone is about 10% lower relative to the BM. The highest hardness is in the stir zone due to recrystallization.
2. The average hardness value of the metal weld zone for the MIG and TIG techniques using ER4043 have a lower hardness value than the heat-affected zone (HAZ) and base metal (BM) due to the differences in their main elements where the filler material ER4043 is Al-Si.
3. The ultimate tensile strength (UTS) of the FSW tensile specimen has been found to be 80% higher than that of the MIG and TIG tensile specimens.
4. FSW welds show the highest efficiency, around 97%, compared to 54% and 55% for MIG and TIG welds, respectively.
5. The place of fracture of the tensile tested specimens obtained by all welding procedures are in the weld metal for TIG and MIG welded specimens, i.e., in the stir zone for the FSW specimen.

## Figures and Tables

**Table 1:** Chemical properties of base material AA 2024-T351.

Alloying Element	Mn	Fe	Mg	Si	Cu	Zn	Ti	Al
wt.%	0.65	0.17	1.56	0.046	4.7	0.11	0.032	Balance

**Table 2:** Mechanical properties of base material AA 2024 T351.

Yield Strength *YS* (MPa)	Ultimate Tensile Strength *UTS* (MPa)	Elongation at Break *E* (%)	Hardness HV
370	481	17.9	137

**Table 3:** Chemical composition of the filler material of wire EN ISO 18273 [20] S Al 4043A (AlSi_5_).

Element	Mn	Fe	Mg	Si	Cu	Zn	Ti	Be	Al
wt.%	<0.15	<0.6	<0.2	4.5–5.5	<0.3	<0.1	<0.15	<0.0003	Balance

**Table 4:** Welding parameters for the MIG butt joint welding process.

Welding Process	Run	Current I (A)	Voltage U (V)	Welding Speed v (cm/min)	Heat Input H = I·U·η/v (J/mm)
MIG	1	155	21.7	41.88	385
	2	180	23.2	52.92	379
	3	170	22.7	49.98	371
TIG	1	230	12.9	11.55	1233
	2	240	11.7	17.93	751.3
	3	200	12.6	12.09	998
	4	200	13.3	20.47	624

**Table 5:** Friction stir welding parameters [18,19].

Sample	Rotation Rate n rpm	Welding Speed v mm/min	Ratio n/v rev/mm
A-I	750	73	10.27
B-II		116	6.47
C-III		150	5

**Table 6:** Elements’ concentrations for MIG joint sample (%).

Spectrum Label	Spectrum 4	Spectrum 5
Al	42.78	55.23
Si	2.15	3.23
Mn		8.64
Fe		12.68
Cu	55.07	20.21
Total	100.00	100.00

**Table 7:** Elements’ concentrations for TIG joint sample (%).

Spectrum Label	Spectrum 12	Spectrum 13	Spectrum 14	Spectrum 15	Spectrum 16	Spectrum 17
Mg	0.18	0.21	1.49		0.23	0.20
Al	35.65	26.70	16.09	59.44	26.66	98.10
Si	62.48	70.24	80.71	9.98	71.38	0.98
Mn				12.94		
Fe				15.07		
Cu	1.68	2.85	1.72	2.56	1.72	0.71
Total	100.00	100.00	100.00	100.00	100.00	100.00

**Table 8:** Tensile test results of welded joints.

Welding Process	Yield Strength *YS* (MPa)	Ultimate Tensile Strength *UTS* (MPa)	Elongation at Break *A* (%)	Joint Efficiency %
MIG	209	261	1.3	54
TIG	145	263	5.2	55
FSW	336.6	469.09	7.2	97

## Data Availability

The original contributions presented in the study are included in the article, further inquiries can be directed to the corresponding author.
